# Targeting Epigenetic Aberrations in Pancreatic Cancer, a New Path to Improve Patient Outcomes?

**DOI:** 10.3390/cancers10050128

**Published:** 2018-04-28

**Authors:** Brooke D. Paradise, Whitney Barham, Martín E. Fernandez-Zapico

**Affiliations:** 1 Schulze Center for Novel Therapeutics, Division of Oncology Research, Department of Oncology, Mayo Clinic, Rochester, MN 55905, USA; Barham.Whitney@mayo.edu (W.B.); fernandezzapico.martin@mayo.edu (M.E.F.-Z.); 2 Mayo Clinic Graduate School of Biomedical Sciences, Mayo Clinic, Rochester, MN 55905, USA; 3 Medical Scientist Training Program, Mayo Clinic, Rochester, MN 55905, USA

**Keywords:** pancreatic cancer, epigenetics, chromatin structure and dynamics, pharmacologic inhibitors, tumor reprogramming

## Abstract

Pancreatic cancer has one of the highest mortality rates among all types of cancers. The disease is highly aggressive and typically diagnosed in late stage making it difficult to treat. Currently, the vast majority of therapeutic regimens have only modest curative effects, and most of them are in the surgical/neo-adjuvant setting. There is a great need for new and more effective treatment strategies in common clinical practice. Previously, pathogenesis of pancreatic cancer was attributed solely to genetic mutations; however, recent advancements in the field have demonstrated that aberrant activation of epigenetic pathways contributes significantly to the pathogenesis of the disease. The identification of these aberrant activated epigenetic pathways has revealed enticing targets for the use of epigenetic inhibitors to mitigate the phenotypic changes driven by these cascades. These pathways have been found to be responsible for overactivation of growth signaling pathways and silencing of tumor suppressors and other cell cycle checkpoints. Furthermore, new miRNA signatures have been uncovered in pancreatic ductal adenocarcinoma (PDAC) patients, further widening the window for therapeutic opportunity. There has been success in preclinical settings using both epigenetic inhibitors as well as miRNAs to slow disease progression and eliminate diseased tissues. In addition to their utility as anti-proliferative agents, the pharmacological inhibitors that target epigenetic regulators (referred to here as readers, writers, and erasers for their ability to recognize, deposit, and remove post-translational modifications) have the potential to reconfigure the epigenetic landscape of diseased cells and disrupt the cancerous phenotype. The potential to “reprogram” cancer cells to revert them to a healthy state presents great promise and merits further investigation.

## 1. Introduction

Pancreatic cancer has among the highest mortality rates of cancer in the world, killing 43,090 people in 2017 alone [1]. It is estimated that more than 95% of these pancreatic cancer cases are pancreatic ductal adenocarcinomas (PDACs), making it the most common and most severe form of pancreatic cancer [2]. Unlike pancreatic endocrine tumors which often have a benign behavior, PDAC originates from exocrine pancreas [2]. Due to the aggressive nature of the disease, the five-year survival rate for metastatic PDAC is a meager 8%. Even for those non-metastatic cases, the survival rate is only 29% [1]. About half (52%) of the 53,670 newly diagnosed cases in the past year were already at a distant stage, lowering their survival rate to just 3% [1,3]. There are many factors contributing to the devastating prognosis. Most immediate is the lack of effective treatment for the disease. In addition, pancreatic cancer is aggressive and it is difficult to diagnose early due to lack of biomarkers of the disease and delayed manifestation of symptoms in patients [3]. Many patients who present with symptoms of the disease have already reached stage 4. Currently, our therapeutic strategies for late stage pancreatic cancer are mostly centered around symptom management instead of a cure. With its aggressive nature and high mortality rate, novel therapeutic strategies to fight pancreatic cancer are urgently needed. 

Historically, the development of pancreatic cancer was attributed solely to mutations in DNA. More recently, we have come to understand it as a much more complex, multi-factoral disease arising from both genetic and epigenetic aberrations. Pancreatic cancer, in particular PDAC, is hallmarked by commonly mutated genes such as *KRAS*, one of the most well-known being a constitutively active mutant form, *KRAS^G12D^* [4]. Therapeutics against genes like *KRAS* and downstream effectors have shown little success, and this may be explained in part by the presence of other mutations and the recent discoveries regarding the involvement of epigenetics in pancreatic cancer development and progression. Current studies have attributed the rapid progression of the disease to epigenetic changes such as DNA methylation alterations and histone tail modifications [5,6,7]. Epigenetic changes such as these allow cells to alter the expression of genes without changing the DNA code itself. Epigenetic modifications provide a rapid and dynamic response to environmental changes in a manner that is reversible and does not affect the underlying genetic code. These changes can lead to silencing of important tumor suppressor genes or cell cycle checkpoints as well as hyperactivation of oncogenes and growth stimuli [8]. These same epigenetic changes that allow for cellular adaptation to an environment can also confer resistance to therapeutic drugs after exposure for extended periods of time. 

Although the involvement of epigenetic regulation in pancreatic cancer presents an additional level of complexity, it also provides an exciting new window for therapeutic opportunity. These alterations in epigenetic pathways can result in differential gene expression in cancer cells and non-cancer cells present in the tumor microenvironment. The reversible nature of these epigenetic modifications offers the potential to reset the epigenetic landscape to that of what it was before the onset of disease. This idea of tumor reprogramming is novel and heavily based in the emerging fields of epigenetics. Preliminary studies have shown the advantages of epigenetic reprogramming in increasing drug responsiveness, altering tumor microenvironment, “resetting” the phenotype of cancer cells to one that is benign [9,10,11,12,13,14]. 

**Figure 1:**
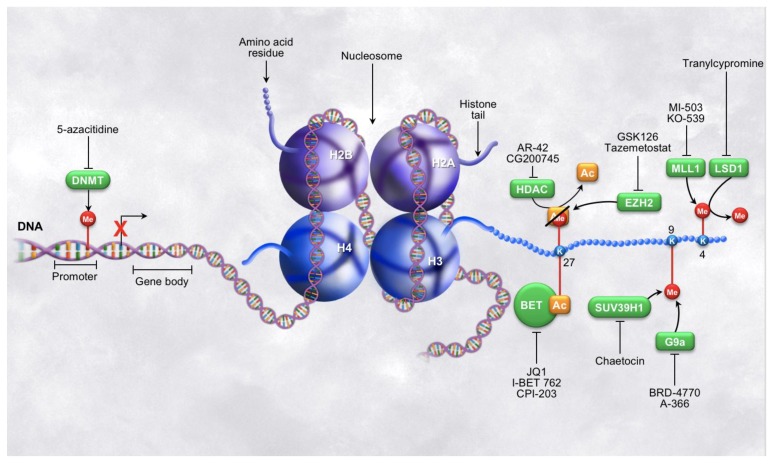
Epigenetic modifications on DNA and histone tails. Illustrations of various epigenetic post-translational modifications, the enzymes that write and read the modifications, as well as a brief summary of some of the inhibitors of the epigenetic regulators currently in use. The left side of the figure depicts epigenetic regulation at the level of DNA methylation and the right side of the figure depicts epigenetic regulation at the level of histone tail modifications. Note: the modification shown on the top side of K27 represents two different marks that may appear on this lysine residue at a given time, not a hybrid state.

Common epigenetic modifications such as DNA methylation and histone post-translational modifications are in the spotlight of epigenetic therapeutics and are increasing in promise as a cancer treatment (Figure 1). DNA methyltransferases have been used as novel cancer therapeutic strategies mostly due to their robust responses to inhibitors credited to the intrinsic reversible nature of the methylation marks [15,16]. Numerous studies have established the aberrations in DNA methylation in all types of cancer cells including PDAC and the significance in driving disease. Many of these marks function to silence important tumor suppressors, such as p16, as well as compromise genomic integrity both of which contribute to pancreatic cancer development and progression [17,18,19]. Histones have become of increasing interest within the past decade because most histone modifying enzymes act only on one or a select few histone marks to either place or remove the modification on the histone tail (writers or erasers) or recognize the specific modification (readers). Thus, they have become ideal enzymes to focus on for use in targeted therapy. Loss of tri-methylation on histone 3, lysine 9 (H3K9) increases metastatic progression in pancreatic cancer [20]. Similarly, H3K27me3, is altered in many types of cancer and has been associated with poor outcome in pancreatic cancer patients [21,22,23]. Another mark of interest that has been correlated with poor outcome is H3K4me3. Alterations in this mark have been shown to mediate PDAC tumor formation as well as immune evasion [24,25]. Increased activity of histone deacetylases (HDAC) is common in pancreatic cancer and can lead to decreased histone acetylation modifications which in turn leads to gene repression. This is particularly harmful in cancer as many downregulated genes are tumor suppressors such as p27 and p53 [26]. Reader proteins have come to light more recently as therapeutic targets, especially the bromodomain and extra-terminal domain (BET) family of chromatin adaptors. These bromodomain-containing proteins can recruit transcription factors to the DNA after interacting specifically with the acetylated lysine residues of the histone tails, further enhancing the transcriptional activation resulting from the acetylation mark. In this way, BET proteins contribute to the growth of PDAC cells utilizing the epigenetic landscape [27]. This wide array of aberrant epigenetic marks are suitable targets for novel therapeutic strategies and show promise for the development and use of epigenetic enzyme inhibitors for cancer treatment and tumor reprogramming. The third leg of epigenetic therapeutics is focused on the targeting potential of miRNAs. Altered endogenous miRNA expression has recently been linked to aberrations in gene expression in pancreatic cancer cells driving disease progression, cell migration, and metastasis [28]. miRNAs can also function as tumor suppressors and are often repressed in cancer cells. Many miRNAs have been linked to the onset and progression of pancreatic cancer, and enhancing the specific miRNA activity within the cell has potential to become a widely used therapeutic approach to prevent disease progression. The Food and Drug Administration (FDA) approved therapy, Miravirsen, uses miRNA in the treatment of hepatitis C and has encouraged the pursuit of miRNA-based therapeutics in pancreatic cancer treatment [29]. Unfortunately, to date, no strategies involving miRNAs or the similar siRNAs have been tested in clinical trials for the treatment of pancreatic cancer, and miRNA will not be extensively covered in this review, but we encourage individual research into current progress with miRNA investigations (Table 1) [28,29,30,31,32]. 

In this review, we will discuss preclinical efforts targeting pancreatic cancer epigenetic aberrations and the challenges in this field. This includes pharmacological efforts to decrease the activity of writer and reader enzymes for aberrant epigenetic marks to elicit anti-proliferative effects within cancer cells (Table 1). We will also cover attempts to reset the epigenetic landscape to that of a healthy cell using the same inhibitors, thus increasing cancer susceptibility to existing therapeutic regimens.

## 2. Targeting Epigenetic Pathways as a Way to Reprogram Tumor Biology

For almost every epigenetic mark that has been correlated to disease, there is a pharmacologic inhibitor for the enzymes that write or read that mark. These inhibitors are effective and have high targeting efficiencies. Their effects can be seen both at a molecular modification level as well as throughout the pathways and cellular phenotypes they affect. The reversible nature of these marks is what inspires the idea of tumor reprogramming. This new idea of tumor reprogramming is innovative and holds tremendous potential as a cancer therapeutic. The use of drugs to rewrite a chromatin landscape is a concept that is already in play in various fields centered around epigenetics. Much of the current research in stem cell therapy and regenerative medicine makes use of epigenetic drugs to manipulate cells and elicit a desired phenotype [33,34]. These fields have experienced tremendous success and more importantly, have shed light on the potential to translate these concepts into PDAC research. 

The reprogramming capabilities of these drugs have arisen within the field both to alter the malignant and harmful phenotype of a cancer cell to that of something benign as well as a means to reprogram the microenvironment of pancreatic tumor making them more susceptible to therapeutics [35]. Drugs that inhibit epigenetic regulators have been used to prevent tumor cells from driving phenotypic changes in the surrounding cells, inhibiting the formation of the stroma that characterizes PDAC [36,37]. Additionally, they have been used to induce an alternate differentiation state in the cells giving them an entirely different phenotype in various types of cancers [14,38]. Research making use of these epigenetic inhibitors to reprogram cells is cutting edge and much is left to come to fully utilize the potential of these drugs. The concept of tumor reprogramming via epigenetic manipulation extends past general therapeutics and into the realm of personalized medicine. Using this approach for therapy could open the door to tailor cancer treatment to target the exact aberrations of a single patient and more effectively eradicate their disease. Tumor reprogramming could be part of a powerful combination therapy or effective on its own. With continued research, we have the potential to offer the field both a versatile and personalized therapeutic. 

## 3. Targeting DNA Methylation

Current research of DNA methylation is focused on the efficacy of DNA methyltransferase (DNMT) inhibitors for cancer treatment. More specifically, studies have highlighted the advantages these epigenetic drugs offer in decreasing proliferation and sensitizing cancer cells to existing radiation and chemotherapies. Cai et al. noted that there are critical threshold levels of DNA methylation within a cancer genome that set it apart from that of comparable healthy tissues. In their work, they uncovered an achievable threshold of methylation to stay below that can be reached with DNMT inhibition. This approach revealed that successful inhibition of DNMT1 via binding a hemimethylated DNA strand during replication will prevent the daughter cells from receiving the full methylation state of the parent cells, ultimately decreasing the levels of DNA methylation in the new generation of cells [39]. Many potent inhibitors of DNMTs are readily available and are being widely used in cancer research today. One of the most common pharmacologic inhibitors of DNMTs is 5-azacitidine, which is a cytosine analog that intercalates into DNA and binds DNMTs, trapping it after one round of DNA replication [40]. Treatment using azacitidine has proven to be effective in many cases as both an anti-proliferative and a sensitizing agent. Cohen et al. found the use of azacitidine to be particularly effective as a sensitizing pre-treatment to nanoparticle chemotherapy in patients with advanced or metastatic solid tumors. They found it was effective in decreasing methylation of target regions of the cancer genome that had been silenced through DNA hypermethylation [10]. Common regions that are silenced in the cancer genome include important tumor suppressors and cell cycle checkpoint machinery [17,18]. This could explain the effectiveness of DNMT inhibitors in decreasing cancer cell proliferation. Additionally, azacitidine has been found to be effective in sensitizing pancreatic cancer cells to ionizing radiation [9]. Although efficacious, using 5-aza as a DNMT inhibitor can have off target effects due to the global demethylation caused by the vast spectrum of gene targets that each enzyme possesses. In addition, 5-aza has been shown to have toxicity as many DNA intercalating agents do, which emphasizes the need for the next generation of DNMT inhibitors. 

In an attempt to decrease the risk of toxicity with the use of current DNMT inhibitors, researchers have turned to identification of new compounds as well as combinational therapies. Foundational studies in silico have uncovered potential DNMT inhibitors with less toxicity than the traditional DNA intercalating agents. Krishna et al. identified three less toxic DNMT1 inhibitors that avoid intercalation into the DNA but still bind to the DNMTs as a ligand of the enzyme. These compounds were tested in vitro and showed anti-proliferative effects in breast cancer and showed promise for investigations that are still underway [41]. Maleimide derivatives of RG108 act as non-nucleoside inhibitors and have been used as another way to address the toxicity of agents like 5-azacitidine. The potency of these drugs has been directly correlated to cytotoxicity on certain cancer lines, but they pose a different issue. The activity of these agents within cancer cells is low, and their mechanism of action is unknown, making it a difficult task to improve the efficacy of these inhibitors [42]. There have also been efforts to design completely new inhibitors, with the intention of increasing their half-lives as well as to identify more effective ways to target those inhibitors specifically to tumor DNMTs while leaving the healthy cells unaffected. Clinical trials employing these epigenetic inhibitors as combination therapies with chemotherapy or radiation as treatment for PDAC have recently emerged. The challenge with these types of clinical trials lies in the aggressive nature of the disease. That in combination with the amount of time it takes to reprogram an epigenetic landscape means many of these studies do not reach completion [37]. Similarly, DNMT inhibitors have also been increasing in popularity as part of combination therapies with HDAC inhibitors. In some breast cancer samples, the combination of DNMTi with HDACi increased ERα mRNA in ERα-negative breast cancer cells lines and consequently decreased proliferation of the cancer cells when subsequently treated with tamoxifen [40,43]. These combination therapies are now being used with the intentions of reprogramming tumor cells to increase their sensitivity to chemotherapeutic agents, as mentioned previously. Some recent efforts have uncovered the potential of using combinations of DNMT inhibitors with HDAC inhibitors in restoring the expression of important tumor suppressor genes such as p15 and mitigating the deleterious cancer phenotype [44]. Considering the recent results in the field, DNMT inhibitors hold great promise for cancer therapeutics in the future, especially with the aim of tumor reprogramming in mind.

## 4. Targeting Histone Modifications

The increased specificity of small-molecule inhibitors targeting enzymes responsible for histone tail modifications has allowed for promising epigenetic-based pancreatic cancer therapeutics [45]. Aberrant H3K9me3 is a common signature of pancreatic cancer and is most often an indicator of gene repression. Histone methyltransferase, G9a, is one of the major enzymatic writers of this mark and has become a popular target for inhibition in pancreatic cancer. G9a works as a member of different complexes; consequently, there are different ways to inhibit the activity of G9a within the cell. G9a most often acts in complex with G9a-Like Protein (GLP), but also acts on a variety of other epigenetic writers such as Polycomb Repressive Complex 2 (PRC2) to coordinate its activity, and has been targeted as a member of these different complexes [46]. G9a is hyperactive in many PDAC cells, making it a viable target for inhibition in cancer treatment. 

Inhibition of H3K9 methyltransferases has been successful as a monotherapy, producing encouraging anti-tumor results in vitro. G9a inhibitors have been shown to induce apoptosis as well as decrease proliferation or cell viability in many cancer types characterized by overexpression of G9a, including pancreatic cancer [47]. Furthermore, pharmacological and siRNA mediated inhibition of G9a activity triggered heightened levels of autophagy in pancreatic cancer cells, ultimately lowering cell viability [48]. The small molecule inhibitor, BRD-4770 has high specificity for G9a over other methyltransferases and resulted in decreased H3K9 methyl marks, decreased proliferation via G_2_/M cell cycle arrest, and increased cellular senescence [49,50]. Yuan et al. experimented with the active metabolite of BRD-4770 as a more potent inhibitor of G9a and found that it was even better at inhibiting G9a activity, but this compound has not been followed since [49]. Another compound, A-366, has been used as an inhibitor of G9a and GLP in leukemia and was effective in increasing differentiation of cancer cells as well as inhibiting proliferation [51]. The G9a inhibitor, BIX-01294, induced apoptosis and ER stress as well as decreased proliferation on multiple occasions in vitro in pancreatic and other cancer cell models [47,49]. Various preliminary approaches to decrease the function of G9a in pancreatic cancer models have shown that this epigenetic regulator is a suitable target for therapeutics and should be further explored. 

G9a inhibition alone has shown success as a cancer therapeutic, but is even more effective as part of a combination therapy. It has been used both as a means to re-sensitize chemotherapy resistant cells and as a combination to target multiple epigenetic aberrations downstream of their oncogenic drivers. Pharmacologic inhibition of G9a using UNC0638 as well as genetic knock-out of G9a in pancreatic cancer models has been shown to increase sensitivity to gemcitabine as well as decrease cancer stemness [52]. Treatment with UNC0638 decreased levels of G9a protein and reduced tumor growth in vivo. Not surprisingly, levels of lysine demethylase, KDM7A, and E cadherin were also increased while polycomb-like 3 (PCL3) was decreased, all of which are regulated by the absence or presence of G9a, respectively. What is significant about this particular finding is the elucidation of the relationship between G9a and KDM7a, which is an epigenetic regulator of E cadherin. Increased activity of KDM7A is correlated with higher expression of E cadherin which in turn is correlated to fewer cancer cells and less epithelial-mesenchymal transition, migration, and invasion [46]. 

Another histone tail modification frequently dysregulated in PDAC is hallmark H3K27 mono- and tri-methylation. Enhancer of Zeste Homolog 2 (EZH2), the catalytic subunit of PRC2, is responsible for depositing this methyl mark. EZH2 is a fundamental and necessary contributor to pancreatic cancer cell stemness [53]. Both EZH2 and its associated PRC2 complex proteins have efficacious small molecule inhibitors that work independently as well as synergistically to reduce cancer cell proliferation and tumor growth in various types of cancers. Inhibitors such as CPI-1205 have been shown to selectively inhibit EZH2 and decrease H3K27 tri-methylation marks leading to decreased cell proliferation as well as increased apoptosis. Some such inhibitors have reached clinical trial for B-Cell lymphomas and medulloblastomas, but there is still work to be done with these inhibitors in PDAC [54,55]. Much of the preclinical work being done with EZH2 inhibitors as a monotherapy has shown promise in many types of PDAC models including monolayer and spheroid culture systems as well as patient-derived xenograft mouse models. UNC1999, an EZH2 specific inhibitor, was successful in all three of these model systems, not only reducing aberrant K27 methyl marks that characterize PDAC cells, but also slowing proliferation rates of the cancer cells [56]. Additionally, GSK126, a common EZH2 inhibitor, is an effective treatment both in monolayer systems as well as xenografts in various types of cancers, decreasing proliferation, angiogenesis and increasing apoptosis [57,58]. 

Prolonged exposure to EZH2 inhibitors can confer resistance to the drug in many cancer cell lines. Recent discovery has shown that small molecule inhibitors against different binding domains of the PRC complex apart from EZH2 can have equal effects in the EZH2i desensitized cell lines. One such example is a small molecule, EED-226, that allosterically binds in the EED binding pocket of PCR2 subsequently inhibiting H3K27 tri-methylation by PRC2. Furthermore, these inhibitors can be used in combination to prevent the development of resistance as well as to induce synergistic effects, increasing the efficacy of treatment as compared to monotherapies. This has been seen with the combination of an EZH2 inhibitor, EI1, and EED-226 [59]. Small molecule inhibitors targeting EZH2 and other H3K27 methyltransferases are effective treatments for various types of cancers, as they promote a reduction in H3K27me3 leading to cell cycle arrest and increased apoptosis. 

Combination therapies with other genetic and epigenetic inhibitors instead of chemotherapeutics have also increased the efficacy of H3K9 methyltransferase inhibition. G9a and EZH2 inhibitor combination treatment decreased H3K9 and H3K27 methylation marks, respectively, in breast cancer leading to decreased cell growth and colony formation. The selectivity of the combination treatment was confirmed as none of the potential other methyltransferase targets had significant inhibitory action upon treatment [60]. Similarly, Mathison et al. have shown that inhibition of the H3K9 methyltransferase SUV39H1/2, can reduce the growth of PDAC cells in monolayer cells culture, as well as in spheroids, organoids and grafts in vivo. Combined inhibition of AURKA using MLN8237 and H3K9 methyltransferases using a pan-histone methyltransferase inhibitor, chaetocin, induced mitotic catastrophe and proved to be efficacious in preventing progression of the various aforementioned PDAC models [61]. Gossypol, a chemotherapeutic under investigation, has been used in combination with BRD-4770 to induce autophagy related death [50]. G9a inhibitors have also been used in combination with double strand break inducing agents in p53 deficient cell lines that show decreased sensitivity to G9a inhibitors. Using UNC0638 in combination with DNA double stranded break-inducing agents such as etoposide sensitized tumors to DSB-inducing agents at doses low enough to avoid causing toxicity to non-tumor cells [62]. G9a inhibitors are versatile in that they can be used effectively as a standalone treatment, in combination with chemotherapies as sensitizing agents, and as a means to overcome genetically conferred resistance to DSB-inducing agents.

EZH2 inhibitors have also been used as part of combination therapies as pre-treatment to re-sensitize resistant cells lines. Chemo-resistant cancer cells are a growing problem, and many of them harbor mutations in EZH2 or PRC related genes. Ougolkov et al. noted an increase in the nuclear accumulation of EZH2 in chemo-resistant pancreatic tumor cells as well as lower levels of differentiation of those tumors. Artificial EZH2 depletion led to increased sensitivity of these resistant cell lines to chemotherapeutics such as doxorubicin and gemcitabine [63]. Results of this study and others like it have sparked new investigations into the efficacy of combination therapies involving EZH2 inhibitors in addition to chemotherapeutics. Reversal of H3K27 methylation prior to treatment with chemotherapeutics increases response as opposed to chemotherapeutics alone in pancreatic cancer cell lines. 3-deazanplanocin A, a histone methylation inhibitor mainly acting via EZH2 inhibition, has been used as an H3K27 methylation reversal agent, and when used as a short pre-treatment to nanoparticle delivered chemotherapeutics induced apoptosis in both well and poorly differentiated tumors and both gemcitabine resistant and sensitive tumor cells [64]. Combination therapies have demonstrated a novel utility for H3K27 methyltransferase inhibitors in many types of cancers, including pancreatic. 

Tri-methylation of H3K4 is characteristically an activating mark, and is often abnormal in pancreatic cancer. One of the major histone methyltransferases involved in writing this mark is mixed lineage leukemia 1 (MLL1), which interacts directly with the protein, menin. Pharmacologic inhibition of menin using MI-503 has anti-tumor activity both in vitro and in vivo and it was selective towards hepatocellular carcinoma cells, resulting in decreased cell migration as well as reduction of H3K4 methylation and concurrent gene repression [65]. KO-539 recently received FDA approval as a candidate inhibitor of menin-MLL binding for phase I clinical trials in mixed lineage leukemia [66]. H3K4me3 has also been a target for rescuing a drug sensitive phenotype in some resistant cancer cell lines using CPI-445, a KDM5 inhibitor [67]. This lysine trimethyl mark has also been targeted as a means to reduce immune evasion and alter expression of genes that drive tumor formation. Lu et al. discovered enrichment of H3K4me3 at PD-L1 promoter regions in PDAC cells. This gene is known to mediate immune evasion and inhibition of the histone modification via MLL1 inhibition decreased the expression of PD-L1 and immune evasion. Therefore, the use of this epigenetic inhibitor has potential to increase the efficacy of immunotherapies [24]. MLL1 inhibitors have also been used to decrease pancreatic islet tumor formation through decreasing methylation on the promoter region of insulin-like growth factor 2 mRNA binding protein 2 [25]. This suggests that targeting MLL1 and other epigenetic regulators might be a way to alter epigenetic landscapes within cells to manipulate gene expression. 

Transcriptional activation via epigenetic regulation is not limited to methylation of H3K4; acetylation on H3K27 as well as involvement of transcriptional activating proteins can induce an open chromatin conformation and facilitate gene expression. Epigenetic regulators can directly act as activators by stimulating the opening of chromatin to allow transcriptional access to certain genes in the genome. Two common proteins involved in the relaxation of chromatin are CREB-binding protein (CBP) and p300, and they have recently become of interest as therapeutic targets. Use of a CBP inhibitor, ICG-001, reversed some tumor specific changes in gene expression in the transcriptome of pancreatic cancer cells [68]. Acetylation on H3K27 is also well known to be an activating modification on chromatin facilitating an open conformation, and is dysregulated in many types of pancreatic cancer. Inhibition of histone deacetylases has potential to be an epigenetic therapy for PDAC patients. Chen et al. worked with a novel HDAC inhibitor, AR-42, and noted its potent antitumor activity in pancreatic cancer cells. Cell proliferation was inhibited and characterized by cell cycle arrest in the G_2_ phase. Treatment with this inhibitor also increased the amount of DNA damage in the cell and increased expression levels of p53, which expectedly led to increased apoptosis [69]. Although these inhibitors are exhibiting desired effects within cancer cells, it is important to keep in mind the issues of specificity that revolve around this group of inhibitors. One of the difficulties of drug therapy is targeting specificity, and it is particularly prevalent when using drugs such as HDAC inhibitors. HDAC inhibitors have global repressive effects; they do not target a specific enzyme like many other epigenetic inhibitors such as menin inhibitors. In a similar fashion, CBP and p300 act throughout the genome, their effects cascading throughout the cell. 

HDAC inhibitors have been used successfully both to mitigate therapeutic resistance in cancer cells as well as to manipulate the microenvironment of the tumor cells increasing their sensitivity to standard chemotherapeutics. Fritsche et al. noted that HDAC2 is upregulated in pancreatic cancer cells that acquire resistance to etoposide. Inhibiting HDAC2 with valproic acid in synchrony with etoposide treatment increases apoptosis in resistant cells and restores the etoposide sensitive phenotype [70]. Although effective in cell lines, HDAC inhibitors still have low success rates in clinical trials for pancreatic cancer. One explanation for this is in the tumor microenvironment. Pancreatic cancer’s characteristic fibroblast rich environment gives tumor cells a survival advantage. The dense stroma created by the fibroblasts creates the ideal environment for the formation of a tumor as well as forming a protective layer, decreasing the effectiveness of drug treatment. Nguyen et al. found that prolonged used of HDACs in vivo can actually induce a more aggressive fibroblast phenotype, which in turn supports the growth of the tumor cells. They noted that HDAC2 binds and deacetlyates pro-inflammatory genes, so its inhibition will lead to increased expression of pro-inflammatory genes creating tumor-supportive environments. Upstream inhibition of some of the pro-inflammatory genes mitigated these unintended effects of HDAC inhibitors and restored their efficacy as anti-tumor drugs. Combination therapies such as these may attenuate the aggressive and inflammatory phenotype of these fibroblasts, disrupting the cancer environment and increasing their susceptibility to drug treatment [12]. 

In addition to targeting writers of the histone code, therapeutic agents targeting readers of histone modifications have also shown promise as cancer therapeutics. JQ1 is a small molecule inhibitor of the BET domain found in several bromodomain-containing reader proteins. The bromodomain is responsible for the recognition and binding of acetylated lysine residues, allowing other domains within these proteins to facilitate recruitment of proteins necessary for initiation and elongation to open accessible locations in the genome. By inhibiting this domain with JQ1, PDAC development is suppressed [71,72]. In addition, when combined with an HDAC inhibitor, Mazur et al. were able to augment cell death and suppress PDAC development more effectively, as the two drugs synergize [72]. Another small molecule inhibitor, I-BET 762, has been equally effective in inhibiting the bromodomain and decreasing cell proliferation. This study also shed light on the alterations of the microenvironment that result from treatment with one of these inhibitors. Intentional remodeling of the tumor microenvironment through the use of JQ1 led to the suppression of pancreatic cancer by decreasing the protective stroma formed by the fibroblasts. Suppression of the cancer associated fibroblasts can not only decrease cancer cell proliferation, but also re-sensitize gemcitabine resistant cancer cells [13]. Additional studies have illustrated the importance of the BET family of proteins in various differentiation pathways, further establishing the reprogramming potential of epigenetic drugs. Through the use of bromodomain inhibitor CPI-203, Nakagawa et al. were able to significantly decrease the amount of differentiated intestinal cells without compromising the existing stem cell population in their model [73]. Preventing both the reading and the writing of histone modifications can be an effective cancer therapeutic, both as a standalone treatment and in combination with existing chemotherapies [29,74].

## 5. Challenges in the Field

Current challenges in the translation of the targeting epigenetic aberrations in pancreatic cancer revolve around lack of preclinical models to accurately study epigenetic inhibitors efficacy and determine marker of response. There has been incredible progress in the development of model systems to test drugs, but there is much left to do. As in most cancer fields, the transition from monolayer to in vivo studies is burdened by a high probability of failure to reproduce the results obtained from in vitro experiments. Emergence of organoid culture systems has increased PDAC modeling efficiency, but these systems also have their own challenges. Furthermore, replicating these studies in clinical trial is increasingly difficult due to the aggressive nature of the disease and limited patient survival times. Though we have made significant progress in our modeling systems and patient studies, much is left to do to accurately recapitulate disease epigenetic-driven phenotypes in vitro and study them extensively in vivo.

Along with the challenges posed by the model systems, we also face lack of understanding of epigenetic pathways and their mechanisms. Since epigenetic alterations function as a means for cellular response to environmental stimuli, they are by nature very dynamic pathways. Elucidating the conditions that drive a particular response is challenging, which makes knowing when to use specific epigenetic drugs no more sophisticated than guesswork. The dynamic nature of these pathways adds yet another layer of complexity into the design and use of inhibitors as therapeutics. One such example arises in the ability for the regulators themselves to change the context in which they are active by decreasing dependence on other complex proteins to carry out their function. Some inhibition strategies involve targeting complexes as a whole, or proteins that interact with the epigenetic regulators but not the regulators themselves. Decreasing dependence on protein complexes will also decrease inhibitor efficacy, rendering the therapy ineffective. Furthermore, the underlying mechanism driving the aberrant activation of certain epigenetic regulators is unknown is many cases. Activation or inhibition of these epigenetic pathways could be a result of genetic mutations in the regulators themselves or activation of upstream signaling pathways, increasing the complexity of using targeted inhibitors for therapy. Inhibition of pathways due to inactivating mutations of the epigenetic regulators may provide opportunity for other pathways to be upregulated. For example, if the methyltransferase MLL has an inactivating mutation, this may allow an opposing silencing mark to be deposited by EZH2, increasing its activity. What this means for patient care and therapeutics is that not only do mutations in epigenetic regulators themselves have negative effects, but they may also affect a cascade of regulatory enzymes. This indicates that targeting a single epigenetic regulator that has altered activity may not be sufficient and instead additional regulators responding to the initial change must be targeted. These mutations and responses vary throughout tumor samples, further emphasizing the role of personalized medicine in tailoring epigenetic-based treatments to the individual and maximizing therapeutic efficacy. Elucidation of when, why, and in what context certain epigenetic pathways are activated, as well as improving targeting specificity will improve therapeutic output in this field and help address the aforementioned challenges.

In addition to a need for disease modeling as well as understanding epigenetic pathways, we must improve the pharmacology of the drugs themselves. These inhibitors have been evaluated for their ability to target specific enzymes in vitro, however, the field is lacking in information about the targeting efficiency of these drugs to remodel the epigenome and to reprogram the tumor to impair growth [75]. Some success has arisen in increasing the targeting efficacy of these drugs through use of multifunctional nanoparticles in combination with molecular imaging to guide inhibitors to PDAC cells, but much is left to do in areas such as this [76]. Pancreatic cancer does have characteristic biomarkers that are becoming of increasing interest as a means to increase tumor cell targeting. Although these individual biomarkers are not completely unique to PDAC tissues, they are commonly used to identify PDAC pathologically, and taken together, can create a unique signature in comparison to heathy tissue. Identifying and targeting biomarkers such as mesothelin or urokinase plasminogen activator in combination with insulin growth factors have shown success, but these concepts are new and there is much left to do before bringing these therapeutic strategies into the clinic [77,78,79,80]. Although research has shown time and again that epigenetic drugs are effective in eliciting a desired cellular response, much remains unknown about the mechanism of action and cellular pathways involved in driving that response. Much of this uncertainty may be attributed to the dynamic nature of the epigenetic landscape of a cell, as previously discussed. To improve the use of these drugs as therapeutics, we must elucidate not only the mechanism of action of the drug, but also what cellular environment facilitates that response so we can better understand how and more importantly, when to use these drugs to target cancer with maximal efficiency.

As we reflect on the targeting efficiency of these drugs, we must also consider the biological controversies that have arisen with targeting some of these epigenetic regulators. Some can serve many purposes within a cell and respond to various extracellular signals for gene regulation. A prominent example is the ability of EZH2 to function as a tumor suppressor. Use of small molecule inhibitors has proven efficacious in blocking the activity of many of these epigenetic regulators but we must also consider that these drugs cannot discriminate the intended action of the enzyme within the cell. The activity of the enzyme will be diminished in the cell regardless of the various functions it might be preforming, like that of a tumor suppressor. Many epigenetic regulators can function as co-regulators for various transcription factors, producing global transcription regulatory effects which may become problematic. Without the ability to discriminate between the specific roles of the epigenetic regulators while using the inhibitors, we encounter unintended effects due to the complete blockade of these regulators in all of their contextual activities. Ultimately, we block both the disease promoting hyperactivity, but also block necessary functions such as tumor suppression or transcription factor co-regulation. Considering these biological controversies, using these drugs in vivo may cause more harm than therapeutic benefit unless we are able to elucidate more efficient targeting strategies to deliver the drug specifically to PDAC cells in the appropriate context. However, the diverse array of roles that each epigenetic regulator holds could be the key to advancing therapeutic targeting specificity. The concept of epigenetic modifiers as co-regulators for many different transcription factors increases our opportunity to target specific functions by targeting unique interactions instead of generally targeting the enzyme in all of its activities. We could then target the context in which the aberrant epigenetic regulator is driving a disease phenotype to inhibit disease while maintaining the integrity of the epigenetic regulator in all of its desirable roles. These notions only further emphasize the need for more thorough investigations of drug effects on the entire pathway and not just how well a specific enzyme is targeted. We conclude that investigations of epigenetic therapies for pancreatic cancer are far from complete, but immense progress has been made in recent years in the field showing promise for the advancement of pancreatic cancer treatment. 

## 6. Conclusions

Epigenetic therapeutics are increasing in efficacy in cancer treatment due to our increasing understanding of cancer as both a genetic and epigenetic disease. They suitably target reversible marks in the epigenome, making them some of the first drugs with the capability to “reprogram” cells to a normal phenotype or sensitize drug therapy-resistant cells. We have seen preclinical success in targeting the main enzymes responsible for writing and reading aberrant epigenetic marks but we need to continue to drive therapeutic development forward by improving pancreatic cancer epigenetic model systems, targeting efficiency of the drugs, and elucidating the mechanisms of epigenetic pathways as well as when they are active. Building on the foundation of knowledge currently available will bring us closer to taking full advantage of the incredible therapeutic capacity of epigenetic drugs.

## Figures and Tables

**Table 1:** Summary of epigenetic therapeutics and their targets.

Epigenetic Pathway	Enzymatic Target	Drug Name	Trial/Clinical Setting
DNA methylation	DNMT1/2	5-azacitidine	FDA approved (myelodysplastic syndromes)
	DNMT1	RG-108 derivatives	
H3K4me	Menin (MLL binding)	MI-503	
	Menin (MLL binding)	KO-539	
	KDM5	CPI-445	
	LSD (KDM1A)	GSK2879552	Trial NTC02929498; recruiting
		Tranylcypromine	FDA approved (depression)
H3K9me	G9a	BRD-4770	
		A-366	
		BIX-01294	
		UNC0638	
	SUV39H1	Chaetocin	
H3K27me	EZH2	CPI-1205	Trial NCT02395601; Phase I; accruing
		UNC1999	
		GSK126	
		Tazemetostat	Trial NCT03009344; NCT02860286; both active, not recruiting
	demethylating agent	3-deazaneplanocin A	
H3K27Ac	HDAC	AR-42	Tirals NCT02795819; NCT01798901; NCT01129193; all accruing
		CG200745	Trials NTC02737228; NCT02737462; both recruiting
	CBP	ICG-001	
	BET family	JQ1	
		I-BET 762	
		CPI-203	
miRNA-122	Hepatitis C Virus	Miravirsen	Trial NCT02508090; Phase II; complete

Abbreviations in the table are as follows: histone 3 (H3), lysine 4 (K4), lysine 9 (K9), lysine 27 (K27), methylation (me), acetylation (Ac), DNA methyltransferase (DNMT), enhancer of zeste homolog (EZH2), histone deacetylase (HDAC), CREB-binding protein (CBP).
